# Research on the coupling and coordinated development of Guangxi’s tourism industry, new urbanization and environmental health system in the post-epidemic era

**DOI:** 10.3389/fpubh.2024.1331765

**Published:** 2024-07-12

**Authors:** Qiuli Meng, Hongwen Pi, Yu Nie, Jixian Ma

**Affiliations:** School of Management, Guangxi Minzu University, Guangxi, China

**Keywords:** post-epidemic era, Guangxi, tourism industry, new urbanization, environmental health, coupling and coordination

## Abstract

As one of China’s sunrise industries, tourism has always been the engine to promote the development of the national economy, and in 2018, the annual income of China’s tourism industry exceeded 5 trillion yuan unprecedentedly. In recent years, the traditional extensive production mode has inevitably brought about problems such as environmental pollution and public health threats, while helping the development of new urbanization, thus triggering a series of challenges in the environmental health system. The tourism industry, new urbanization, and environmental health system three cooperate and promote each other, the coordinated development between them for economic growth, new urbanization development, environmental protection, and public health play a vital role, in the post-epidemic era is a special period of historical opportunities, the public’s focus from the original sacrifice of environmental health in exchange for economic growth model began to green, low-carbon sustainable development mode, Guangxi Zhuang Autonomous Region as a tourism resource endowment rich region, It is of positive significance to explore the coupling degree and coordination between the tourism industry, new urbanization and environmental health system, and put forward targeted practical enlightenment, which is of positive significance for promoting the sustainable development of tourism industry. Taking Guangxi Zhuang Autonomous Region as a case study, this paper constructs three comprehensive evaluation index systems of the tourism industry, new urbanization and environmental health system, and analyzes and compares the weights of various indicators in the three fields of tourism economy, new urbanization and environmental health system in Guangxi by using the entropy weight TOPSIS method. The coupling coordination model was used to measure the coupling degree and coordination degree of the tourism industry, new urbanization and environmental health system construction in Guangxi Zhuang Autonomous Region from 2009 to 2021. The empirical results show that the weights of various indicators change with the development of the social economy. The comprehensive efficacy index of Guangxi’s tourism industry has increased year by year for 11 consecutive years; At the end of the evaluation period, after the outbreak of the new crown epidemic, the evaluation index of the tourism industry, new urbanization and environmental health system all showed a downward trend to varying degrees. Before the pandemic, the coupling coordination type of the three subsystems generally experienced a transformation of “moderate dissonance-reluctant coordination-primary coordination-intermediate coordination,” but the overall development level was still poor. After the outbreak of the new crown epidemic, the coupling and coordination between the three has been reduced to a state of poor coordination. Because of the above research conclusions, this study proposes to make full use of the important time node of the post-epidemic era and proposes to actively promote the development of the tourism industry, promote the upgrading of the industrial structure, use digital empowerment of the economic form, optimize the environmental health system and other targeted countermeasures to keep the coupling degree and coordination between the three within a reasonable range. This ensures the sustainable development of social systems in the region. This study has made some contributions to the development of high-quality tourism and a healthy environment. First of all, it enriches the content of the environmental health system. This study takes the ecological environment and atmospheric environment in the environmental health system as the entry point and adds the index content of the environmental health evaluation system, which provides a certain supplement for the relevant research on the environmental health system strength. Secondly, the relationship between the tourism industry, new urbanization and environmental health is analyzed and into a unified theoretical framework. This study takes the Guangxi Zhuang Autonomous Region, which is rich in tourism resources, as a case study site, and innovatively explores the coupling and coordination relationship among the tourism industry, new urbanization and environmental health system in the case site. Finally, it provides targeted countermeasures for the sustainable development path of the three systems of tourism industry, new urbanization and environmental health in the case site in the future. It is of positive practical significance to compare the coupling and coordination degree between the three, realize the coordinated, orderly, and healthy development of the three in the region, and provide operational suggestions for the upgrading of the tourism industry structure, the benign development of new urbanization, and the formulation of environmental health system policies.

## 1 Introduction

At the end of 2019, an outbreak of novel coronavirus pneumonia (“NCP”) occurred quietly and swept the world rapidly. The outbreak of NCCP is not only a “black swan” event in public health but also has a profound impact on air pollution. China has entered the post-epidemic era after an all-out effort by all sectors of society to “fight” the epidemic. It is obvious that environmental issues have become a major problem for all countries in the world, and it has become a consensus to protect and improve the environment and protect public health. How to achieve rapid economic and social development while keeping environmental health risks within an acceptable range requires our continuous efforts in practice. The 14th Five-Year Plan period is an important strategic opportunity period for China’s tourism development. With the opening up to the outside world and the construction of “One Belt, One Road,” China and ASEAN have established a strategic partnership of mutual trust, mutual understanding, mutual benefit, and mutual assistance. Now ASEAN has become China’s largest trading partner, and China-ASEAN relations have become the most successful and dynamic model of regional cooperation in the Asia-Pacific region. Guangxi is at the intersection of the three national strategies of “One Belt and One Road,” Yangtze River Economic Belt, and Beibu Gulf Economic Zone, and has the unique location advantage of “one bay with eleven countries, benign interaction between East and West,” and is an important window of China to ASEAN, and has good tourism resources and development conditions. In the key period of the most active development of Guangxi’s export-oriented economy, the development of the tourism industry as a sunrise industry is particularly important. Industrialization and urbanization are the basic driving forces of China’s sustained economic growth (1). It is important to go deeper within the tertiary industry and explore the construction path of new urbanization with the modern service industry as the core, especially to play and realize the synergistic development between the service industry and new urbanization (2). In recent years, environmental pollution, air quality decline, and ecological damage have also been gradually highlighted, and strengthening environmental protection has become necessary for development and desired by people’s livelihood. Economic development and environmental health are complementary to each other and can be transformed into each other, just as it is often said that “green water and green mountains are golden mountains.” Therefore, it is of certain strategic significance to explore the coupled and coordinated development of the tourism industry, new urbanization, and environmental health in Guangxi, to build a green and sustainable development model and to realize the benign development of the tourism industry.

By reviewing the relevant literature, we found that the development level of the tourism industry in Guangxi Zhuang Autonomous Region is relatively low on the whole, and some counties and cities have imperfect transportation networks and unsystematic protection of resources and environment, etc., which all impose certain restrictions on the development of tourism industry, and also cause a series of problems such as single employment structure and low-income level of residents. Few studies have been conducted in the Guangxi Zhuang Autonomous Region on the coupled and coordinated development of the tourism industry, new urbanization, and environmental health systems. Based on this, this paper uses the entropy-weighted TOPSIS method, which combines the entropy value method and TOPSIS method, to explore the development level of the comprehensive evaluation indexes of the three subsystems of tourism industry, new urbanization and environmental health in Guangxi Zhuang Autonomous Region from 2009 to 2021 using Guangxi Zhuang Autonomous Region as a case study site, aiming to quantitatively and objectively evaluate the development level of tourism industry, new urbanization and environmental health. The aim is to quantitatively and objectively evaluate the development levels and development history of the tourism industry, new urbanization and environmental health. Subsequently, the coupling coordination degree model is adopted to give specific measurement values for quantitative analysis of the coupling coordination relationship among the tourism industry, new urbanization and environmental health systems, thus providing a quantitative reference basis for decision-making on the coordinated development of the tourism industry, new urbanization and environmental health in Guangxi.

The tourism industry is rich in elements, intertwined and combined, constituting a closely integrated industrial chain, which is based on the industries contained in tourism itself and associated with the primary industry, secondary industry, and health, sports, culture and arts, finance, public services and other industries in the tertiary industry (3). The study of the coupled and coordinated relationship between tourism and other industries has a positive significance for promoting the healthy and sustainable development of tourism.

Foreign studies on the relationship between the ecological environment, tourism industry, and urbanization are more mature. The type of research is mostly single-factor analysis of tourism, urbanization and ecological environment, and the research on the correlation between multiple systems is less, and among the research on the correlation between multiple systems, the empirical research on the interaction between two systems is more common, and the research on the interaction between multiple systems is less than the former. Mullins proposed the concept of “tourism urbanization” for the first time in 1991, and subsequently, tourism urbanization has become a hot topic of research for scholars and governments at all levels at home and abroad (4, 5). Mullins argues that the mechanisms of tourism urbanization are mainly manifested in the fact that tourism can cause the expansion of the urban population, the restructuring of urban industries, and the change of urban class structure, especially the emergence of modern services and the prominence of the petty bourgeoisie as the main signs of the influence of tourism on urbanization (6). Thays analyzed the impact of tourism-led urbanization on the ecosystem environment around the Araruma Lagoon in Brazil (7). Ozturk used the environmental Kuznets curve hypothesis to study the interaction between tourism and ecological footprint (8). Izza Mafruhah studied the causal relationship between tourism development and marine ecological sustainability (9). Achmad Fandi researches the role of stakeholders in increasing tourism potential to support tourism industry activities, especially in a dynamic environment. The main objective of this study was to identify the factors that influence the improvement of the tourism industry and to produce strategies for the tourism industry (10). Genjin Sun et al. analyze the impact mechanism of environmental regulation on China’s tourism development from the perspective of the integration of institutional and environmental economics (11). Dan Yuan studied How to coordinate the relationship between tourism and ecology (12). Qin Yang Research on the coupling and coordinated development of the tourism industry and regional economy in the economic circle of the Sichuan-Chongqing region in southwest China (13). Wei Yang studied whether the tourism industry and technological progress affect ecological economic development (14). Yu Zhang analyzed how to coordinate the tourism industry with the ecological environment (15). Muhammad Irfan’s research unleashes the dynamic impact of the tourism industry on energy consumption and environmental sustainability (16). Xiao Yu’s study on coupling coordination of the human settlement environment and tourism industry (17). Fengting Zhang’s research on coupling coordination of the regional economy, tourism industry, and the ecological environment (18). Yufeng Cheng explored the ecological performance of China’s tourism Industry (19). Liranran research on understanding and handling the relationship between the tourism industry, ecological environment, and regional economy correctly (20). Aiqin Ding analyzed the coupling effect measure of the tourism industry and new urbanization (21). Fengtai Zhang analyzed how new urbanization affects tourism Eco-Efficiency in China (22). Ming Hao et al. research a coupling relationship between New-Type urbanization and tourism resource conversion efficiency (23). Xingyu Yang’s research on coupling and interaction between tourism eco-efficiency and new urbanization in the Yangtze River economic belt (24). Dongmin Zhang’s research on uncoordinated coupling assessment of new urbanization and ecological carrying capacity in the Yellow River Basin (25). Shuwang Yang and Jing Li analyzed whether new-type urbanization curbs haze pollution (26, 27). Le Ma’s research on the spatiotemporal evolution of urban carbon balance and its response to new-type urbanization (28). Haitao Wu analyzed whether environmental pollution inhibits urbanization in China (29). Yazhen Zhang’s research on spatial and nonlinear effects of new-type urbanization and technological innovation on industrial carbon dioxide emission in the Yangtze River Delta (30). Kanda Xue studies the coupling coordination between New-Type Urbanization and Water Ecological Environment and its driving factors (31).

A review of the relevant literature revealed that the discussion of the two industrial systems seen through coupled coordination degree models is relatively common in China. For example, Li Mengcheng conducted a BP neural network model measurement and coupled coordination analysis of tourism development and ecological environment in 12 island counties in China (32); Xiong Jianxin analyzed the coupled coordination relationship between tourism and urbanization in the Dongting Lake area (33); Qu Xiaoshang studied the coupled coordination degree between tourism economy and ecological environment in the provincial capitals of the Yellow River Basin region (34); Zhang Changcheng studied the Chengdu-Chongqing region’s coupled coordination between tourism and new urbanization (35). While there are fewer discussions between the three industrial systems, for example, Geng Nana studied the coupled coordination among ecological environment, tourism industry, and urbanization in the Yellow River Basin (36); Gao Dongmei studied the coupled coordination among the three subsystems of tourism, economy, and ecological environment in Chinese provinces (37); Weng Gangmin studied the coupled coordination among tourism, ecology, and urbanization in the Beijing-Tianjin-Hebei region (38); Yang Xiuping studied the coupled coordination relationship of tourism environmental carrying capacity from three subsystems: natural, economic, and social (39); Luo Xue made a coupled coordination analysis of urbanization and ecological environment based on urban functional areas (40); Zhang Jiajie made a study on the coupling degree of ecological environment quality and urban development in Chengdu-Chongqing economic circle based on Google Earth Engine platform (41); Shi Zhiyu analyzed the Yangtze River Delta urban agglomerations and its coupling and coordination with urbanization (42); Wenjia Li studied the local coupling and remote coupling of urbanization and eco-environmental quality based on multivariate remote sensing data (43).

After systematically combing the existing literature, we found that although previous studies have included three systems, tourism, urbanization, and ecological environment, the relationship between them has also shifted from one-way influence research to two-way interaction research, and the use of coupled coordination degree, synergy degree, VAR and other econometric models and grayscale analysis, regression analysis and other research methods to quantitatively present the degree of coordinated development between the systems in different regions, the research. The theoretical basis and data analysis methods have become increasingly mature. However, most of them are still exploring the interaction between two of them or combining other subsystems, and there is still a lack of integration tourism industry, new urbanization and environmental health into a unified theoretical structure, and there is even less research on the coupled coordination relationship among tourism, new urbanization and environmental health systems in Guangxi Zhuang Autonomous Region, the case study of this paper. In conclusion, the study of the coupling and coordination relationship among tourism, new urbanization and environmental health in Guangxi Zhuang Autonomous Region is of great significance, which can better promote the coordinated and healthy development among the three, and thus promote the economic development in the whole region of Guangxi.

## 2 Materials and methods

### 2.1 Constructing the index system of tourism industry, new urbanization and environmental health system

This paper follows the evaluation principles of scientificity, systematization, accessibility, and comparability, and draws on the existing research results (1, 39, 44–46) to determine the following items as relevant industry evaluation indicators. it includes seven evaluation indexes of the tourism industry, 16 evaluation indicators of new urbanization, and 10 evaluation indexes of the environmental health system.

### 2.2 Research method

This paper adopts the entropy weight TOPSIS method as the main method and technique for the comprehensive development level of the tourism industry, new urbanization and environmental health system in Guangxi Zhuang Autonomous Region, and measures the coupling degree and coordination degree among tourism industry, new urbanization and environmental health system in Guangxi Zhuang Autonomous Region through the coupling coordination degree model. To avoid the influence of subjective factors in the process of weight determination, we choose to use the entropy TOPSIS method to assign weights to the system indicators first and use the comprehensive score to represent the system.

#### 2.2.1 Entropy TOPSIS method

##### 2.2.1.1 Entropy TOPSIS method meaning

The entropy method is a mathematical method used to determine the degree of dispersion of a certain indicator. The greater the degree of dispersion, the greater the influence of that indicator on the overall evaluation. The traditional “double basis point method,” also called TOPSIS (Technique for Order Preference by Similarity to Ideal Solution) method, can be used to solve the problem of evaluating and ranking multiple solutions with multiple indicators in the socio-economic field. In the alternative solution set, a set of best indicator data is used as a virtual positive ideal solution, and a set of worst indicator data is used as a virtual negative ideal solution according to the nature and data of the indicators, and the superiority of the evaluated solution is judged by comparing the distance of the solution points from the positive and negative ideal points (46). Its general determination of weights based on expert opinion leads to the evaluation results being susceptible to human subjective factors, the determination of weights using the TOPSIS method is an important link, and the use of the information entropy method can effectively eliminate the influence of subjective factors (47). The entropy TOPSIS method is essentially an improvement of the traditional TOPSIS evaluation method, in which the weights of evaluation indexes are determined by the entropy method, and then the ranking of evaluation objects is determined by the TOPSIS method using the technique of approximating ideal solutions. In this paper, based on the improved entropy method to measure the weights of index order parameters, the TOPSIS method is used to measure the Euclidean distance, to reveal the closeness of the real state of the system to the ideal state, which can objectively measure the comprehensive efficacy index of the three systems of tourism and, new urbanization and ecological civilization system, and then scientifically evaluate the comprehensive development level of tourism and, new urbanization and ecological civilization system.

##### 2.2.1.2 Entropy TOPSIS method-specific steps

The main calculation steps of the entropy TOPSIS method are as follows (47–51):

(1) Assuming that objects are being evaluated and *n* evaluation indexes for each object being evaluated, the judgment matrix is constructed as follows:

(1) $$X=\left(X_{ij}\right)m\times n\;\left(i=1,2,\;\ldots ,\;m;\;j=1,2,\;\ldots \;,\;n\right)$$

(2) Normalization of the judgment matrix.

(2) $$X_{ij}=\frac{X_{ij}}{X_{\text{max}}}\left(X_{\text{max}}\;\text{is}\;\text{the}\;\text{maximum}\;\text{value}\;\text{under}\;\text{the}\;\text{same}\;\text{indicator}\right)$$

(3) Calculate the information entropy.

$$H_{j=-K\sum_{i=1}^{m}P_{ij}}\ln P_{ij}$$

(3) $$\text{In}\;\text{the}\;\text{formula},P_{ij}=\;\frac{X_{ij}}{\sum_{i=1}^{m}X_{ij}};k=\frac{1}{\ln m}$$

(4) The weights of indicator j are determined.

(4) $$\omega_{j}=\frac{1-H_{j}}{\sum_{j=1}^{n}\left(1-H_{j}\right)}$$

In the formula, $\omega_{j}\in \left[0,1\right]$, and $\sum_{j=1}^{n}\omega_{j}=1$.

(5) Calculate the weighted normalization matrix.

(5) $$R={\left(r_{ij}\right)}_{m\times n,}r_{ij=}\omega_{j}\cdot X_{ij}\left(i=1,2,\;\cdots ,\;m\;;\;j=1,\;2,\;\cdots ,\;n\right)$$

(6) Determine the optimal solution $S_{j}^{+}$ and the inferior solution $S_{j}^{-}$.

(6) $$S_{j}^{+}=\max\left(r_{1j,}r_{\;2j,}\cdots ,\;r_{nj}\right),\;S_{j}^{-}=\min\left(r_{1j,}r_{\;2j,}\cdots ,r_{nj}\right)$$

(7) Calculate the Euclidean distance between the evaluation object and the optimal and inferior solutions.

(7) $$sep_{i}^{+}=\sqrt{\sum_{j=1}^{n}{\left(S_{j}^{+}-r_{ij}\right)}^{2}},sep_{i}^{-}=\sqrt{\sum_{j=1}^{n}{\left(S_{j}^{-}-r_{ij}\right)}^{2}}$$

(8) Determine the relative proximity $C_{i}$ to characterize the composite evaluation index:

(8) $$C_{i}=\frac{sep_{i}^{-}}{\;sep_{i}^{+}+sep_{i}^{-}},C_{i}\in \left[0,1\right]$$

Where, the larger the value of $C_{i}$, the better the evaluation object is.

#### 2.2.2 Coupling coordination degree model

Through the analysis of the relationship between the tourism industry, urbanization and environmental health in the previous paper, it is seen that there is a coupling and coordination phenomenon among these three. Referring to the research results of Xiaolin et al. (52), an index system reflecting the structural characteristics of the three is established to quantitatively analyze the coupling degree of the ternary system, and the coupling coordination results are used to judge the coupling coordination degree between tourism industry development, new urbanization construction and environmental health system in Guangxi Zhuang Autonomous Region. System (53), “financial system-industrial structure” system (54), and “innovation capacity-economic development” system (55), In recent years, the model has also been used in tourism research, including the “tourism-ecological environment” system (56), the “tourism-urbanization” system (57) and the “tourism-regional development” system (58).

##### 2.2.2.1 Coupling and coordination model

In this paper, we borrow the concept of coupling degree in physics and refer to the research results of Cong Xiaonan (58) to give the measurement model of coupling degree using the method of characterizing the deviation formula between multiple systems.


(9) $$C_{k}={\left\{\left(U_{1}\times U_{2}\times \cdots U_{k}\right)\prod \left(U_{i}+U_{j}\right)\right\}}^{1∕k}$$

According to Equation (1), the coupling degree model of the triadic system of tourism industry, new urbanization and ecological civilization construction is established:


(10) $$C_{3}={\left\{\left(U_{1}\times U_{2}\times U_{3}\right)/\left(U_{1}+U_{2}+U_{3}\right)\times \left(U_{1}+U_{2}+U_{3}\right)\right\}}^{1∕3}$$

Among them, $C_{3}$ represents the coupling degree, $U_{1},U_{2}\;\text{and}\;U_{3}$ represent the comprehensive evaluation indices of the tourism industry, new urbanization and ecological civilization system, respectively. Since the tourism industry, new urbanization and ecological civilization systems all have different differences in their respective development history (59), to avoid deviating from reality, a coupling coordination degree model (60) is constructed by drawing on the research results of scholars such as:


(11) $$D=\sqrt{C\times T,}\;其中\alpha U_{1}+\beta U_{2}+\gamma U_{3}$$

Where, *D* is the coupling coordination degree of the tourism industry system, new urbanization and ecological civilization city system, *T* is the comprehensive evaluation index of the tourism industry, new urbanization and ecological civilization city, and α,βandγ are coefficients to be determined. Through the calculation of Equations (3)-(11), the coupling coordination among the three systems is eventually determined.

##### 2.2.2.2 Coupled coordination model data pre-analysis processing and partitioning criteria

The data are internalized before analysis, and the internalization formula is: *a* + (*b* − *a*) × (*X* − min)/(max − min), where *b* is 0.99, a is 0.01, max and min denote, respectively, the maximum and minimum values of a term. All the data are between 0 and 1 after the internalization process (Tables 1–3).

**Table 1:** Evaluation Index of the tourism industry and new urbanization.

Subsystem	The name of the metric	Unit	Attribute
Tourism industry	Total tourism spending	100 million yuan	+
	Domestic tourism consumption	100 million yuan	+
	Foreign exchange consumption for international tourism	$10,000	+
	The number of domestic tourists received	10,000 people	+
	The number of inbound tourists received	1	+
	Turnover of accommodation and catering services above the designated size	100 million yuan	+
	Number of star-rated hotels	1	+
The new type of urbanization	Proportion of urban population	%	+
	Population density	Person/per square kilometer	+
	Natural population growth rate	%	+
	Proportion of employed population in secondary and tertiary industries	%	+
	GDP per capita	Yuan	+
	The output value of secondary and tertiary industries accounts for the proportion of total output value	%	+
	Investment in fixed assets per capita	Yuan	+
	Per capita disposable income of urban residents	Yuan	+
	10,000 college students	1 person	+
	Number of health technicians per 1,000 people	1 person	+
	Number of buses for 10,000 people	Vehicle	+
	Postal and telecommunications business volume per capita	Yuan	+
	Built-up area per capita	Square meter	+
	Paved road area per capita	Square meter	+
	The area of garden green space per 10,000 people	Hectare	+
	Green coverage of built-up areas	%	+

**Table 2:** Environmental health system evaluation indicators.

Subsystem	Level 1 indicators	Secondary indicators	Unit	Attribute
Environmental health	Ecological environment	Total amount of industrial wastewater discharged	10,000 tons	−
		Total industrial emissions	Cubic meters	−
		Comprehensive utilization rate of industrial solid waste	%	+
		Harmless treatment rate of domestic waste	%	+
		Green cover area	Hectare	+
		The area of green space in the park	Hectare	+
	Atmospheric environment	Excellent air quality rate throughout the year	%	+
		Annual average concentration of SO_2_	μg/m^3^	−
		Annual average concentration of NO_2_	μg/m^3^	−
		PM10 annual average concentration	μg/m^3^	−

**Table 3:** Classification standard of coupling coordination degree.

Coupling coordination degree *D* value interval	Level of coordination	Degree of coupling coordination
[0.0–0.1]	1	Extreme dissonance
[0.1–0.2]	2	Serious dissonance
[0.2–0.3]	3	Moderate dissonance
[0.3–0.4]	4	Mild disorder
[0.4–0.5]	5	On the verge of disorder
[0.5–0.6]	6	Barely in tune
[0.6–0.7]	7	Primary coordination
[0.7–0.8]	8	Intermediate level coordination
[0.8–0.9]	9	Good coordination
[0.9–1.0]	10	Quality coordination

The coupling degree is determined by the subsystem’s integrated sequential covariates, which characterize how closely tourism, urbanization and environmental health interact with each other. When *C* = 0, it means that the coupling degree is very low, the subsystems are irrelevant to each other, and the system develops in a disorderly way; when *C* = 1, it means that the coupling degree is very high, the subsystems are closely cooperating, and the system moves in an orderly way. Using the median segmentation method, when *C* ∈ [0, 0.3], the system is in the low-level coupling stage; when *C* ∈ [0.3, 0.5], the system is in the fly-down grinding stage; when *C* ∈ [0.5, 0.8], the system is in the benign coupling stage; when *C* ∈ [0.8, 1], the system is in the high-level coupling stage.

### 2.3 Data sources

The data in the paper mainly come from Guangxi Zhuang Autonomous Region 2009–2021, Guangxi Statistical Yearbook, National Economic and Social Development Bulletin, and Ecological Environment Bulletin.

### 2.4 Empirical analysis

According to Equations (1) and (2), SPSS was used to process the basic data by means of the mean value in the standardized processing method, and then the weights of the evaluation indicators of the three subsystems of the tourism industry, new urbanization and environmental health from 2009 to 2021 were calculated (Tables 4–6).

**Table 4:** Weights of tourism industry evaluation indicators.

Serial number	1	2	3	4	5	6	7	8
Evaluation indicators	Proportion of urban population	Population density	Natural population growth rate	The proportion of the employed population in the secondary and tertiary industries	Area of paved roads per capita	GDP per capita	Built-up area per capita	Landscaping area per 10,000 people
Weight	0.0035	0.0002	0.0168	0.0165	0.0099	0.4573	0.0074	0.0050
Serial number	9	10	11	12	13	14	15	
Evaluation indicators	The output value of secondary and tertiary industries accounts for the proportion of total output value	Investment in fixed assets per capita	Per capita disposable income of urban residents	10,000 college students	Number of health technicians per 1,000 people	Number of public cars per 10,000 people	Post and telecommunications business per capita	
Weight	0.0002	0.1379	0.0216	0.0339	0.0187	0.0130	0.2582	

**Table 5:** Weight of evaluation indicators of new urbanization.

Serial number	1	2	3	4	5	6	7	8
Evaluation indicators	Proportion of urban population	Population density	Natural population growth rate	The proportion of the employed population in the secondary and tertiary industries	Area of paved roads per capita	GDP per capita	Built-up area per capita	Landscaping area per 10,000 people
Weight	0.0035	0.0002	0.0168	0.0165	0.0099	0.4573	0.0074	0.0050
Serial number	9	10	11	12	13	14	15	
Evaluation indicators	The output value of secondary and tertiary industries accounts for the proportion of total output value	Investment in fixed assets per capita	Per capita disposable income of urban residents	10,000 college students	Number of health technicians per 1,000 people	Number of public cars per 10,000 people	Post and telecommunications business per capita	
Weight	0.0002	0.1379	0.0216	0.0339	0.0187	0.0130	0.2582	

**Table 6:** Weight of environmental health evaluation indicators.

Serial number	1	2	3	4	5
Evaluation indicators	Total amount of industrial wastewater discharged	Total industrial exhaust emissions	Comprehensive utilization rate of industrial solid waste	Harmless treatment rate of household waste	Green cover area
Weight	0.3826	0.0907	0.0230	0.0018	0.0699
Serial number	6	7	8	9	10
Evaluation indicators	Park green space	Excellent air quality rate throughout the year	SO_2_	NO_2_	PM10
Weight	0.1920	0.0015	0.2165	0.0102	0.0118

#### 2.4.1 Comprehensive system efficacy index

In this paper, based on the entropy method, the TOPSIS method is combined with the results of the entropy method, and the entropy TOPSIS method is used to calculate the relative proximity through the Euclidean distance, so as to reveal the proximity between the real state and the ideal state of the system, and thus more objectively measure the comprehensive efficacy index of tourism, new urbanization, and environmental health subsystems, and more scientifically It can reflect the comprehensive development level of tourism, new urbanization and environmental health. According to Table 7, from an overall perspective, the comprehensive efficacy index of tourism subsystem in Guangxi showed a rising trend year by year during the 10 years from 2010 to 2019, with an average growth rate of 26.87% and a good development momentum, and the tourism industry was hit hard by the New Crown epidemic in 2020, and the comprehensive efficacy index of tourism also decreased by 52.07% compared with 2019, and in 2021, because the In 2021, the epidemic is effectively controlled, tourism industry has warmed up and increased by 15.19% compared with 2020; the comprehensive efficacy index of Guangxi’s new urbanization subsystem fluctuates up and down between 2009 and 2013, but the overall change is not significant. 2014–2020 increases year by year, with an average increase of 75.33 in 7 years, especially in 2020, the growth rate reaches 356.16%. It indicates that in the early period of the evaluation period, the foundation of new urbanization construction in Guangxi was slightly weak, and the development was constrained and restricted to a certain extent, while with the advantage of late development, the overall development process of new urbanization in Guangxi began to accelerate in the middle and late periods of the evaluation period, reflecting that the policies related to new urbanization construction introduced and implemented in Guangxi were implemented favorably, and the urbanization efficacy index in 2021 was 9.13% lower than that in the urbanization efficacy index in 2021 is 9.13% lower than that in 2020, indicating that the development of urbanization has slowed down due to a series of factors such as the new crown epidemic; the comprehensive efficacy index of environmental health subsystem in Guangxi showed a decreasing trend between 2009 and 2016, with an average decrease of 21.62%. 2017–2019 gradually rebounded, with an average increase of 41.29% in the 3 years. This reflects that due to the successive introduction of national policies and strong governance at the government level, the construction of ecological civilization in Guangxi has achieved a certain degree of success in recent years. 2020–2021 the efficacy index of the environmental health subsystem decreased slightly compared with 2019, but the overall fluctuation is small.

**Table 7:** Tourism industry, new urbanization, ecological environment system entropy weight TOPSIS results and coupling coordination degree results.

Systematic evaluation indicators		Annual												
		2009	2010	2011	2012	2013	2014	2015	2016	2017	2018	2019	2020	2021
Comprehensive efficacy index of the tourism industry	Relative proximity	0.121	0.154	0.204	0.246	0.288	0.335	0.390	0.472	0.581	0.757	0.989	0.474	0.546
Comprehensive efficacy index of new urbanization	Relative proximity	0.032	0.042	0.039	0.048	0.048	0.055	0.066	0.071	0.085	0.136	0.203	0.926	0.081
Comprehensive efficacy index of ecological and environmental systems	Relative proximity	0.723	0.703	0.502	0.515	0.416	0.303	0.240	0.107	0.124	0.145	0.277	0.214	0.240
Results of coupling coordination calculations	Coupling degree *C* value	0.195	0.425	0.579	0.716	0.756	0.815	0.786	0.76	0.726	0.808	0.801	0.462	0.442
	Harmonized index *T* value	0.293	0.273	0.343	0.378	0.426	0.42	0.428	0.448	0.515	0.579	0.697	0.565	0.537
	Coupling coordination *D* value	0.239	0.341	0.445	0.52	0.567	0.585	0.58	0.583	0.611	0.684	0.747	0.511	0.487
	Coordination level	3	4	5	6	6	6	6	6	7	7	8	6	5
	The degree of coupling coordination	Moderate outrange	Mild disorders	On the verge of imbalance	Barely coordinated	Barely coordinated	Barely coordinated	Barely coordinated	Barely coordinated	Junior coordination	Junior coordination	Intermediate coordination	Barely coordinated	On the verge of imbalance

#### 2.4.2 Coupling degree analysis

##### 2.4.2.1 Analysis of coupling results from 2010 to 2014

According to Table 7, within the 5 years from 2010 to 2014, the system coupling degree of “tourism-new urbanization-environmental health system” at the Guangxi district level increased year by year, indicating that in these 3 years Guangxi district level “tourism-new urbanization-environmental health” in the growth process of low-level-high-level coupling, and the three subsystems gradually reach a benign high-level coupling state among themselves in the 5 years.

##### 2.4.2.2 Analysis of coupling results from 2015 to 2019

In the 5 years of 2015–2019, the system coupling degree of Guangxi district-level “tourism-new urbanization-environmental health system” decreases in fluctuation, but the decrease is not significant, and *C* ∈ [0.7–0.8] in 5 years, which indicates that in these 5 years Guangxi district-level “tourism-new urbanization-environmental health New urbanization-environmental health” is in a fluctuating phase of friction and high-level coupling.

##### 2.4.2.3 Analysis of coupling results from 2020 to 2021

In 2020–2021, the coupling between tourism-new urbanization-environmental health at the district level in Guangxi shows a decreasing trend, with a 42.32% decrease in 2020 and an average 4.33% decrease in 2021. The tourism-new urbanization-environmental health system has certain differences in terms of internal structure, functional positioning, and development speed, but there is a correlation between the three.

##### 2.4.2.4 Comprehensive coupling results and cause analysis

The study of the results of coupling degree analysis reveals that tourism, new urbanization and environmental health subsystems in Guangxi do reflect a closely linked coupling relationship as a whole, and imply a strong influence relationship and high interaction intensity between tourism, new urbanization and environmental health. Tourism, as an effective industrial support for new urbanization, is a useful attempt to explore the new urbanization in multiple ways, and the concepts of ecological and environmental quality, social integration and harmony, and advanced lifestyles embodied in the new urbanization development create the necessary conditions and opportunities for tourism development. In addition, according to the table, the coupling degree of the “tourism-new-urbanization-environmental health” system in Guangxi also presents alternating changes of different degrees and modes. The reason is that the development of tourism in Guangxi is changing from traditional sightseeing tourism to health and leisure tourism, and the urbanization reform is just at an important historical stage of “overcoming difficulties.” In addition to the other factors that affect the development of regional economic volume, the running-in smoothness between the evolution of each subsystem and the changes affected by external factors leads to a certain degree of fluctuations in the coupling degree of the system. From 2009 to 2021, the average coupling degree of the “tourism-new urbanization-environmental health” system in Guangxi was 0.636, indicating that the average coupling degree of the three subsystems was basically in the stage of integration during the evaluation period. However, in 2014, the coupling degree of the “tourism-new urbanization-environmental health” system reached the optimal state of 0.815, while the coupling degree of 2009 was the lowest state of 0.195. Taking 2014 as the cut-off point, the average coupling degree of the three subsystems in the region increased progressively from 2009 to 2014. The average coupling degree shows a progressive upward trend in 2009–2014, but in 2015–2019, it begins to show a slight decline accompanied by fluctuations, and a significant decline in 2020. In 2009–2014, the average composite efficacy index of the “Tourism-New Urbanization-Environmental Health” subsystem in the region increased at a low rate of −0.061, while the average efficacy index of the three systems increased from 2016 to 2019, with an average increase of 0.273. Among them, the tourism subsystem showed an increasing trend from 2010 to 2019, with an average efficacy index of 0.4416; the urbanization subsystem composite efficacy index fluctuated up and down from 2009 to 2013, with little overall change; it gradually increased from 2014 to 2020, with an average increase of 0.7533 over 6 years; the environmental health subsystem composite efficacy index showed a decreasing trend from 2010 to 2016, with an average decrease of −0.061. The average change in the composite efficacy index of tourism, new urbanization, and environmental health subsystems in the periods of 2009–2014 and 2015–2019 is also a major factor in the change of coupling degree. In contrast, the harm caused by the new crown epidemic in early 2020 to all sectors, especially tourism, is the direct cause of the significant decrease in the coupling degree in 2020.

#### 2.4.3 Coordination degree analysis

##### 2.4.3.1 Analysis of results

According to Table 7, on the whole, from 2009 to 2019, the coordination degree of the “tourism-new urbanization-environmental health” system in Guangxi shows an increasing trend year by year, with an average coordination degree of 0.537 and an average increase of 0.0508, indicating that the overall operation of the “tourism-new urbanization-environmental health” system in Guangxi is within the benign growth process of moderate disorder-intermediate coordination. The overall operation of the “tourism-new urbanization-environmental health” system is within the benign growth process of moderate disorder-medium level coordination. The change characteristics of the “tourism-new urbanization-environmental health” system coordination degree, which is a comprehensive index reflecting both the development degree and coupling degree of the tourism-new urbanization-environmental health subsystem, reveal that as the comprehensive development level of tourism, new urbanization, and environmental health in Guangxi gradually improves, the coupling degree of the three systems will increase in the 10 years from 2009 to 2019. Ten years, the coupling degree of the three gradually increases, and the coordination degree also achieves a benign transformation of moderate disorder – mild disorder – near disorder – barely disorder – preliminary coordination – intermediate coordination. From the comparison of the changes in system coordination degree and coupling degree, it can be seen that the changing pattern of the coordination degree is roughly the same as that of the coupling degree until 2020, but at the beginning of 2020, the coordination degree and coupling degree simultaneously undergo a significant decline and remain consistent with the change of development degree. This indicates that it is affected by a series of persistent adverse effects brought about by the new crown epidemic in late 2019, and also reflects the relatively strong influence of the integrated development level of tourism and new urbanization on the system coordination degree. In the post-epidemic era, the development level of each subsystem will continue to rebound as the new crown epidemic is transformed into regular management of category B. At this time, if we further expand the space for improving the system coordination, we should pay sufficient attention to improving the bridging of the subsystem coupling relationship to achieve a benign coordination state among the three.

##### 2.4.3.2 Cause analysis

**Figure 1:**
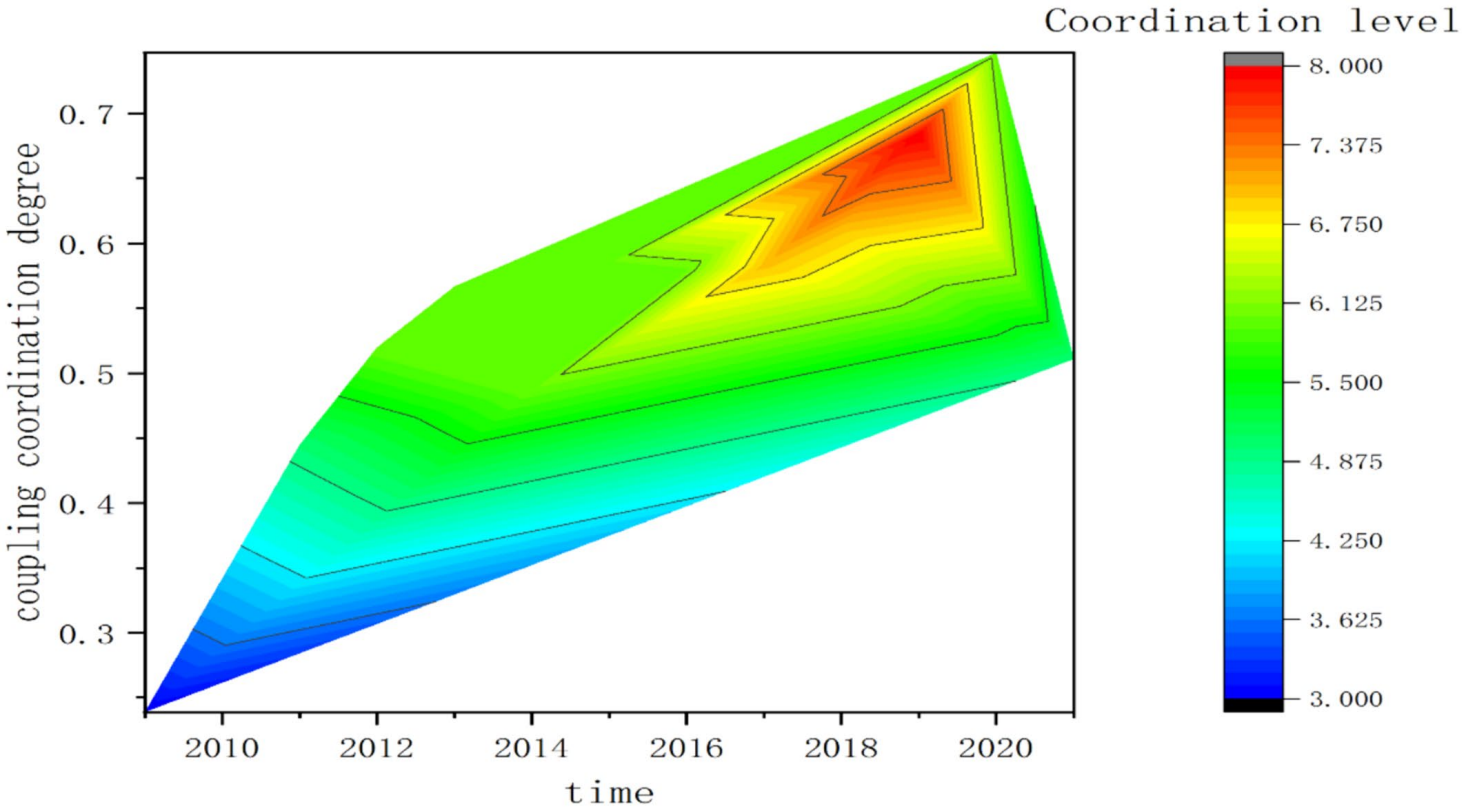
The trend of coupling coordination degree and coupling coordination level of tourism industry-new urbanization-environmental health subsystem in Guangxi Zhuang Autonomous Region from 2009 to 2021.

In the pre-evaluation period, the construction of new urbanization is in the exploration period, and the constraints it faces are varied and complex, involving a wide range of factors. Although there is a cross-sectional gap between tourism and new urbanization in terms of comprehensive development, according to the trend chart analysis (Figure 1), with the deep expansion of tourism and the release of energy level accumulation, the positive effect of tourism on new urbanization is continuously strengthened, contributing to the steady increase of the comprehensive development level of new urbanization. The development gap between the two gradually decreases, thus promoting the benign development of the coupling relationship and ultimately enhancing the coordinated interaction effect of the system. At the early stage of evaluation, the regional tourism industry in Guangxi is relatively weak in terms of development conditions, operational efficiency, and supporting guarantee, and the supply factors created by tourism to the new urbanization are not yet fully utilized, so the coordinated development level of the three subsystems in the whole region is low. In the later stage of the evaluation, as the new urbanization process progresses, its derivative demand for tourism development gradually grows, so the new urbanization, with its significant feed-back mechanism to tourism, accordingly widens the space of interaction and matching between the two, thus ensuring the dynamic improvement of the coordinated development of the system.

## 3 Results

The article takes the Guangxi Zhuang Autonomous Region as the research object, and takes the data from 2009 to 2021 as the research sample, through the index weight index of the three subsystems of the tourism industry, new urbanization and environmental health using the entropy weight TOPSIS method to derive the efficacy index of the three subsystems, on this basis, the coupling coordination degree model is used to quantitatively explore the coupling degree between Guangxi tourism industry, the coupling degree and its coordination level among the tourism industry, new urbanization and environmental health of Guangxi were explored quantitatively based on the coupling coordination degree model, and the following conclusions were drawn.

Firstly, the integrated development and virtuous cycle among the three subsystems of the tourism industry urbanization-environmental health can promote the integration within the three industries and finally achieve the sustainable development goal of mutual benefit and win–win.

Second, during the evaluation period, the weights of the indicators of the three subsystems of the tourism industry, new urbanization and environmental health in Guangxi Zhuang Autonomous Region have been changing with the socio-economic development: in the early period of the evaluation period, except for the tourism industry, which has been increasing year by year, the subsystems of new urbanization and environmental health have decreasing and fluctuating trends to different degrees; in the middle of the evaluation period, the evaluation indices of the three subsystems have shown good growth trend.

Third: before the outbreak of the new crown epidemic in late 2019, the coupling coordination types of the three subsystems of tourism industry, new urbanization and environmental health have generally experienced a “moderate disorder – barely coordinated – primary coordination – intermediate coordination” benign transformation, and in 2020–2021 the coordination degree is affected by the new crown epidemic and turns to a state of near disorder. Although the benign shift was shown before the epidemic, the overall development level is still poor.

Fourth, later in the evaluation period, the mutual integration among the three subsystems of the tourism industry, new urbanization and environmental health is reduced to a barely coordinated stage by the epidemic and other factors. In the post-epidemic era, there is still a long way to go to enhance the coupling and coordination between the three and to allow the integration and progress of the industries to develop together.

## 4 Discussion

To further deepen the development of tourism industry changes, accelerate the promotion of new urbanization construction, and actively promote the construction of ecological civilization based on vigorously maintaining the original ecology; at the same time to further strengthen the linkage of the three, fully create the “1 + 1 + 1 > 3” spillover effect, so that the three present a positive degree of coupling and coordination to promote the tourism industry, new urbanization and ecological civilization. The new epidemic, China’s economic growth rate, and environmental health are constantly being tested, and the interaction between various factors has brought serious challenges to the sustainable development of China’s tourism industry, new urbanization, and environmental health, and in the post-epidemic era, all industries have ushered in new development opportunities:

First, promote the low-carbon transformation of the industry and help upgrade the industrial structure.

In the field of new urbanization construction, from the government level, first, it should strengthen the control of energy consumption intensity and quantity, curb the blind development of high energy-consuming and high-emission industrial projects, and promote the optimization and upgrading of industrial structure; second, it should enhance the effect of energy utilization efficiency by implementing the amplitude saving of the secondary industry mainly in industry and construction, which can be done through the introduction of various award policies or tax incentives, etc. to Encourage localities to increase the consumption of renewable energy; third, it is necessary to coordinate the construction of new urbanization and the development plan of the tourism industry, avoid homogeneous development, eliminate the intensification of market competition caused by the waste of resources, and create favorable conditions for the formation of the core competitiveness of the tourism industry. Traditional industries are not the same as backward industries, as long as they continue to work on the word “reduction” and remove the label of high energy consumption and high emissions, they can be better integrated into the sustainable development process of the tourism industry and ecological environment.

Second, digital empowers the economy and creates new-quality productivity.

At present, the digital economy has penetrated one, two, and three industries, with intelligent technology as the engine of the new technology movement that is sweeping all walks of life. The most significant feature of the digital economy era is undoubtedly digitalization. Digital technology not only changes the traditional business model but also uses big data and Internet technology to enable a comprehensive industrial chain transformation of the traditional industrial economy, creating a deep integration of the digital economy and the traditional real economy. Starting from the government level, we should pay attention to the policy support and precise capital investment of the tourism industry, deepen the application of the digital economy in the tourism industry, and create new quality productivity, which can promote the structural upgrading of the tourism industry in a considerable period and drive the rapid development of the regional economy. At the same time, the construction of new urbanization also requires the development level of regional service industries, including tourism, to be upgraded, to boost development with domestic demand and enhance people’s experience and identity by providing better services, thereby accelerating the construction of new urbanization and promoting the benign and coordinated development of the three.

Third, focus on green development goals, and maintain the sustainability of the environmental health system.

To promote healthy and coordinated development between the tourism industry, new urbanization, and environmental health, we should implement precise measures to promote the development level of the regional service industry. The improvement of the development level of the regional service industry will drive the development of the tourism industry and attract more tourists, which requires that the ecological environment should also be further improved to meet the growing demand of tourists for an ecological environment. Promote the integrated development of new urbanization, environmental health, and tourism industry with the concept of all-for-one tourism development, explore the depth and breadth of the environmental health system, and maintain the sustainability of the environmental health system. The regional economy and tourism industry are closely related to each other, and the ecological environment is the basis and long-term driving force for the development of the regional economy and tourism industry. China has been advocating the concept of a “community of human destiny,” calling on all countries in the world to reduce disagreements, fight the epidemic together, protect the environment together, seek common development, and share the fruits. In the post-epidemic era, it is necessary to seize the favorable opportunity of the initial improvement of the ecological environment and make full use of the ecological environment to support and rely on the regional economy. “Green water and green mountains are the silver mountains of gold,” a firm new development concept, fundamentally prevents the destruction of the ecological environment, increases the punishment for the destruction of the ecological environment, tries to maintain the original ecological environment, reduces automobile emissions, advocate new energy vehicles, maintain a good quality of the atmosphere, tourists will also be with the overall environmental health of the tourists will also travel and consume many times with the benign change of the whole environmental health, thus bringing more opportunities and positive orientation for the development change of tourism industry; meanwhile, the fundamental improvement of environmental health quality will also become the label of new urbanization construction.

## 5 Limitations and prospects

Based on the analysis of the tourism industry, new urbanization and environmental health system in Guangxi Zhuang Autonomous Region mainly from the perspective of economics, this study has some shortcomings in the details.

First, indicators of the tourism industry, new urbanization and environmental health systems. This study involves the construction of the three indicators in the index system and the selection of measurement methods and refers to the methods of some scholars, but the current academic community has not formed a consistent view on the construction and measurement methods of different indicator systems, and different scholars have different treatments and interpretations of related issues. Due to the complexity and diversity of the environmental health system, there are still some indicators that have not been included in the evaluation scope of the indicator system, and the process of empirical analysis, the quality of the indicators often determines the accuracy of the analysis results, so there may be a certain degree of imperfection in the measurement of the indicators of the environmental health system.

Second, in terms of the choice of empirical methods, the entropy-weighted TOPSIS method and the coupling coordination model in quantitative analysis were used for analysis. In future research, the introduction of qualitative analysis, machine learning, and other methods may be able to increase the breadth of research argument perspectives.

Third, in addition to studying the coupling and coordination relationship between the tourism industry, new urbanization and environmental health system, there are still more systems that can be discussed, and they all have an impact on the overall development of the regional economy. In future research, we can not only study the relationship between more systems but also conduct more in-depth research on the subsystems in the tourism industry, such as catering, accommodation, transportation, etc., to increase the depth and comprehensiveness of the research.

## Data availability statement

The datasets presented in this study can be found in online repositories. The names of the repository/repositories and accession number(s) can be found in the article/supplementary material.

## Ethics statement

The studies involving humans were approved by Guangxi University for Nationalities. The studies were conducted in accordance with the local legislation and institutional requirements. The participants provided their written informed consent to participate in this study. Written informed consent was obtained from the individual(s) for the publication of any potentially identifiable images or data included in this article.

## Author contributions

QM: Writing – original draft, Writing – review & editing. HP: Data curation, Methodology, Software, Writing – review & editing. YN: Writing – original draft, Writing – review & editing. JM: Writing – original draft, Writing – review & editing.
